# Developmental enamel defects: a must-know for orthodontic practice

**DOI:** 10.1590/2177-6709.30.2.e25spe2

**Published:** 2025-05-30

**Authors:** Marco Aurélio Benini PASCHOAL, Gabriele ANDRADE-MAIA, Letícia CRISTINE-SILVA, Aniely Ferreira NOGUEIRA, Felícia MIRANDA, Daniela GARIB

**Affiliations:** 1 Federal University of Minas Gerais, Dental School, Department of Pediatric Dentistry (Belo Horizonte/MG, Brazil).; 2 Federal University of Minas Gerais, Dental School, post-graduate Program in Dentistry (Belo Horizonte/MG, Brazil).; 3 University of São Paulo, Dental School, Department of Orthodontics (Bauru/SP, Brazil).

**Keywords:** Developmental enamel defects, Orthodontics, Review, Defeitos de desenvolvimento do esmalte, Ortodontia, Revisão

## Abstract

**Introduction:**

Developmental Enamel Defects (DED) pose a significant challenge for clinicians, particularly orthodontists. These defects can lead to difficulties in differential diagnosis, orthodontic appliance adhesion, treatment planning, and overall patient management.

**Objective:**

The present review aims to provide orthodontists with a comprehensive understanding of DED and their implications for orthodontic treatment. A systematic literature search was conducted to identify relevant studies on DED and their orthodontic management.

**Results:**

The available evidence, primarily from laboratory-based studies, is of low to moderate quality. The most challenging DED cases involve structural loss, especially in molars severely affected by molar-incisor hypomineralization (MIH) and certain types of amelogenesis imperfecta.

**Conclusions:**

To address the limitations of current research, well-designed clinical studies are needed to investigate various aspects of DED management, including pre-treatment of affected enamel, adhesive techniques for bracket bonding and removal, extraction of molars affected by MIH, and interdisciplinary collaboration among dental specialists. By advancing the understanding of DED and refining treatment strategies, orthodontists can improve the outcomes for patients with these conditions.

## RESUMO

**Introdução:**

Os defeitos de desenvolvimento do esmalte (DDE) representam um desafio significativo para os cirurgiões-dentistas, particularmente para os ortodontistas. Desafios como a dificuldade no diagnóstico diferencial, na adesão do aparelho ortodôntico, no planejamento do tratamento e na gestão global do paciente.

**Objetivo:**

A presente revisão da literatura tem como objetivo fornecer aos ortodontistas uma compreensão abrangente dos DDE e suas implicações para o tratamento ortodôntico.

**Métodos:**

Foi realizada uma pesquisa sistemática na literatura para identificar estudos relevantes sobre DDE e tratamento ortodôntico.

**Resultados:**

As evidências disponíveis, principalmente de estudos laboratoriais, são de qualidade baixa a moderada. Os casos mais desafiadores de DDE envolvem perda estrutural, especialmente em molares severamente afetados pela hipomineralização molar-incisivo (HMI) e certos tipos de amelogênese imperfeita.

**Conclusões:**

Para abordar as limitações da presente investigação, são necessários estudos clínicos bem concebidos para investigar vários aspectos do manejo dos DDE, incluindo o pré-tratamento do esmalte afetado, técnicas adesivas para colagem e remoção de braquetes, extração de molares afetados por HMI e colaboração interdisciplinar entre especialistas. Ao avançar na compreensão dos DDE e ao refinar as estratégias de tratamento, os ortodontistas podem melhorar os resultados para os pacientes com essas condições.

## INTRODUCTION

As the prevalence of dental caries has decreased over the decades, structural losses of enamel, whether of chemical origin (e.g., erosive tooth wear), behavioral (e.g., bruxism, tooth erosion), or environmental/local/genetic (e.g., enamel defects), have become easier to detect and thus diagnose. The latter group in particular is closely related to dental caries, since the morphology of the enamel leads to a greater accumulation of biofilm and a lower mineral content, which favors fractures. These aspects can lead to greater episodes of hypersensitivity, restorative failures due to reduced adhesion to the different restorative materials, which has a direct impact on the reduced quality of life related to oral health (especially in terms of functional aspects). 

The orthodontist, when confronted with such an oral problem in its different phenotypes (e.g., quantitative or qualitative defects), may have difficulty in making the appropriate diagnosis, which can lead to doubts about the clinical management, including the most appropriate type of bracket, the most appropriate adhesive technique, the bracket removal technique, the types of polishing after removal, and the proper management of severely affected first molars. Thus, the aim of the present study was to provide relevant information on the different types of enamel defects and how to manage them in orthodontic practice.

### TOOTH ENAMEL

The human body is formed from a single cell, which leads to a complex and coordinated process called embryogenesis. This process involves a series of metabolic activities, which give rise to the different tissues, organs, and systems that make up the body, allowing the body’s activities to function. The embryonic processes result in the formation of the tooth germ, which is made up of structures originating from the ectoderm, responsible for the formation of tooth enamel, and the mesoderm, which gives rise to the pulp, dentin, cementum, and supporting structures.¹ 

**Figure 1:**
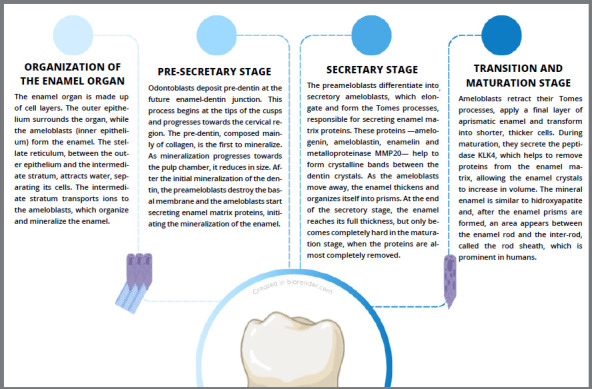
Amelogenesis process.

Teeth are formed by means of odontogenesis, which is divided into different phases. Each phase is responsible for the origin of one of the structures that make up the dental element.² Amelogenesis is the phase of odontogenesis in which enamel is formed and is divided into different stages, as described in Figure 1.

Furthermore, amelogenesis is a complex process, and alterations in the different phases of this process can lead to quantitative and/or qualitative outcomes in enamel structure. Healthy enamel is characterized by a highly mineralized content, with around 96% inorganic matter composed of hydroxyapatite and the remainder, in organic matter and water. The hydroxyapatite crystals are strongly bonded within the enamel prisms, which are organized and distributed perpendicularly from the amelodentinal junction to the outer surface.^³^ These characteristics are responsible for guaranteeing the physical, thermal, and mechanical properties of enamel.⁴ It is therefore essential to understand the characteristics of defective enamel and the challenges involved in dental treatment in these cases. 

### ENAMEL DEVELOPMENT DEFECTS

Considering that tooth enamel is the hardest structure in the human body, it plays a crucial role in providing a wear-resistant layer for the dental crown, protecting teeth against thermal, physical, and chemical forces that can be damaging to the underlying pulp tissue^5^. Unlike other hard tissues present in the body, enamel is not capable of undergoing remodeling or regeneration processes. As a result, any pathological alteration that occurs during its formation becomes permanent, manifesting itself as flaws in the structure, known as Developmental Enamel Defects (DED).^6^

Ameloblasts are the cells of the enamel organ responsible for the formation and maturation of tooth enamel during a physiological process, tightly regulated and marked by intense cellular activity, called amelogenesis. Hereditary, systemic, acquired and local etiological factors may be associated. Alterations such as fever, medication, infections, trauma, hypoxia, hereditary factors and others that occur during any stage of amelogenesis may disturb the proper enamel morphology.^5^ ^,^ ^7^

Therefore, the etiology of DED is multifactorial, as it is a long process that begins in the third trimester of pregnancy until around the age of 18 (when the third molars erupt in the oral cavity), it is not possible to determine which events are associated with it; however, it is possible to have suspicions based on the chronology of tooth formation and the patient’s medical history. The formation period of some teeth coincides, but not necessarily all of them are affected, or equally affected by the condition.^5^ ^,^ ^6^

The phenotype and severity of DED vary according to the stage of development that the enamel organ is in during the alteration, and the intensity and duration of the stimulus are also influencing factors.^5^ Clinically, these defects are visible as they modify the appearance of tooth enamel, presenting themselves in different forms, depending on the stage of development the tooth is in: when in the pre-secretory and secretory phases of the enamel matrix, they can result in quantitative defects, seen as flaws in the tooth’s structure (hypoplasia); when they occur in the maturation phase, they can result in a qualitative deficiency, which is visualized as a change in translucency, with stains or opacities (hypomineralization).^7^ ^-^ ^9^

### TYPES OF ENAMEL DEFECT

#### Dental fluorosis

The incorporation of fluoride ions into tooth enamel occurs physiologically during the process of enamel development and mineralization, and even after the tooth erupts. This interaction occurs through environmental exposure to the ions (fluoridated water, toothpaste, fluoridated salt, professional use of fluoride). The continued topical presence of the ions in the oral cavity is essential, as it acts directly on the De-Re process, making the enamel more resistant to demineralization and, consequently, to dental caries.^10^

However, excessive and chronic fluoride intake during critical periods of the amelogenesis process can result in an irreversible pathological condition characterized by enamel hypomineralization, fluorosis.^5^ ^,^ ^11^ This condition is characterized by increased porosity, with loss of enamel translucency and increased enamel opacity.^10^

The fluoride ion acts directly on the ameloblasts, on the developing protein matrix and on the processing of this matrix, altering the release of protons during mineralization and how these are manipulated during pH regulation. All these effects cause a dose-dependent response to excess fluoride, which generates changes in the morphology and spacing of enamel crystals, resulting in lower mineral content in the enamel.^12^

The severity of fluorosis is determined by the amount of fluoride present in the bloodstream, and everyone has different risks and resistance to developing the condition, based on genetic factors and health conditions.^5^ ^,^ ^13^ In mild cases, the enamel shows fine horizontal white lines, forming opaque grooves that run across the tooth. In more severe cases, on the other hand, the enamel can be colored from milky white to yellow or brown, becoming so porous that it breaks easily, resulting in depressions that usually take the form of cavities. In severe situations, these changes can manifest as a snowflake-like appearance, showing the severity of the enamel damage.^10^ ^,^ ^11^

#### Molar-incisor hypomineralization

Another enamel defect is molar-incisor hypomineralization (MIH), which affects one or all the first permanent molars, and is often associated with permanent incisors. It is a qualitative defect, resulting from inadequate mineralization of the enamel matrix, in which the affected areas have an increased protein content and a decreased mineral content, with less distinct prismatic sheaths and enamel with a lack of organization of the enamel crystals. Therefore, they are more porous and irregular.^5^ ^,^ ^8^ ^-^ ^9^

In its milder form, the enamel shows opacities ranging from white to yellow and brown. Teeth affected by severe MIH can have manifestations ranging from post-eruptive fractures to tooth loss, due to the condition. These teeth are often very sensitive to thermal and mechanical stimuli, as the enamel layer is not adequately mineralized.^5^ ^,^ ^14^

#### Amelogenesis imperfecta

Amelogenesis imperfecta is a hereditary condition of non-syndromic origin, caused by mutations in several genes and mainly affecting tooth enamel. In this defect, a deficient protein matrix leads to hypoplasia, deficient enamel crystal growth and mineralization leads to a decreased level of maturation and mineralization, or abnormal initiation of enamel crystals with subsequent abnormal mineralization or hypocalcification, whereby the enamel has a higher amount of proteins compared to normal enamel, and a hypoplastic and/or hypomineralized phenotype.^5^ ^,^ ^15^ Clinically, enamel can be discolored, sensitive and prone to disintegration, due to post-eruptive breakdown. 

#### Enamel hypoplasia

As mentioned above, dental enamel defects can be quantitative or qualitative. Quantitative defects are the result of alterations in the initial stage of enamel formation, during the secretion of the protein matrix. Thus, some alterations such as malnutrition, infections, local trauma, metabolic stress, genetic and environmental factors can lead to quantitative enamel defects, called enamel hypoplasia.^16^

Since ameloblasts are considered biological markers, the possible factors associated with hypoplastic defects can be identified by means of a detailed anamnesis, favoring guidance for patients and their families. One of the etiological factors related to hypoplasia is vitamin D deficiency. In these cases, a linear and generalized defect is observed, usually symmetrical.^5^

In addition, the main characteristic of hypoplasia is a reduction in the thickness and depth of the enamel. Depending on its etiology (local, systemic, genetic or environmental), it can manifest itself in a localized or generalized way, with an irregular, dotted appearance, or by means of grooves and depressions in the enamel. There is also a common misunderstanding of the correct terminology of defects, which can lead to misdiagnosis and affect clinical management^5^.

**Figure 2:**
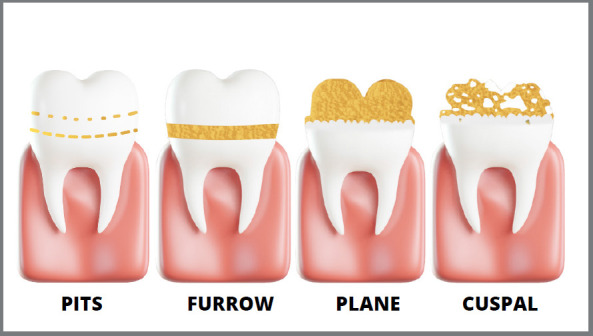
Different clinical aspects presentation of hypoplasia defect.

The Figure 2 illustrates the main clinical manifestations of hypoplasia. It is essential to consider the limits and edges of the defect, the aesthetic impairment, the extent and the number of teeth involved. 

#### Quality of life in patients with DED: clinical challenges

DED particularly in more severe forms, can impact the oral health-related quality of life for both patients and their families, due to functional, aesthetic, and social issues ^17^ ^,^ ^18^. In adolescents, especially when located in the anterior region, the coloration of DED can pose psychosocial challenges, leading to feelings of shame, bullying, low self-esteem, distress, and decreased social interaction.^19^ ^-^ ^21^

Some DED can generate different sensitivity levels in response to thermal and mechanical stimuli, even when the enamel is clinically intact. This makes hygiene, eating and dental care difficult, leading to increased caries experience, impaction, food selectivity and changes in chewing patterns.^21^ ^,^ ^22^ However, some resources can be used to minimize symptoms, including potassium nitrate-based toothpastes, casein phosphopeptide-based prophylactic pastes, low-power laser in analgesic doses, fluoride and bioactive varnishes. 

In addition, hypomineralization-type defects have a higher inorganic content, which makes it difficult for restorative materials to adhere. This can result in a repetitive cycle of restorations, representing a major challenge, including for cementing orthodontic attachments.^20^ ^,^ ^23^ In addition, dentists face the greater resistance of these teeth to anesthesia, possibly due to the presence of subclinical pulp inflammation. In this context, the administration of anti-inflammatory drugs, such as ibuprofen, one hour before the appointment is recommended.^24^

### ORTHODONTIC TREATMENT FOR PATIENTS WITH DED

During the orthodontic management of patients with DED, the professional faces significant challenges due to the particularities of each defect, which can directly influence the planning, choice of appliance, maintenance, and quality of treatment. In addition, the characteristics of malocclusions must be carefully assessed, regardless of the presence of DED, since genetic and environmental factors can be associated with changes in skeletal and dental patterns. Treatment may also be affected by factors such as: the patient’s age, adherence to treatment and patient cooperation, the type of skeletal and/or dental discrepancy and socioeconomic status.^25^ ^,^ ^26^

It should be stressed that regardless of the diagnosis of DED, preventive and interventional strategies during childhood should be encouraged, with the aim of reducing the consequences and minimizing the negative impacts of malocclusions on patients’ growth and development. However, the indication for these interventions and the ideal timing must be considered to achieve the goal and success of the treatment. In addition, depending on the type and severity of the defect, patients may have an extensive dental history, which can have significant financial and psychological impacts.^26^ ^,^ ^27^

In addition to the challenges inherent in orthodontic diagnosis and the planning of complex cases, another relevant aspect to be considered is the adhesion of orthodontic accessories to dental substrates with defects, especially quality defects. In cases in which the mineral composition is compromised, it may be necessary to adopt specific protocols, such as deproteinization, before acid etching the enamel. A multidisciplinary approach is encouraged, emphasizing effective communication between the professionals involved, especially in cases where the patient has undergone restorative treatment before orthodontic intervention. In this context, orthodontists must understand the properties of restorative materials so that they can manage the case appropriately.^28^ ^,^ ^29^

In the amelogenesis imperfecta (AI), the first aspect to consider is the type/subtype or classification of the defect, since it may be a defect that affects the quantity or quality of the enamel of all the dental elements. The approach will be defined according to the degree of the defect, the extent, the associated symptoms, the presence of dental anomalies, and the type of malocclusion to be corrected.^30^ ^,^ ^31^ Also, patients with AI may be more prone to Class II, discrepancies in vertical craniofacial growth, greater intermaxillary angle, decreased overbite and mandibular retrognathism. 

Concerning the types of malocclusions, patients with AI may have a higher prevalence of anterior open bite, making orthopedic intervention ideal in childhood. When intervention in childhood is not possible, orthodontic or surgical strategies may be necessary.^31^ As this condition is not very prevalent, it is important to encourage research and publications on therapeutic approaches in this scenario. 

In cases of dental fluorosis, the severity of the defect can affect orthodontics, especially in fixed appliance treatments. The bond strength of the bracket, which is essential in these cases, can be compromised by the fluorotic dental substrate.^33^ ^,^ ^34^ ^,^ ^35^ Some studies suggest prolonging the acid etching time, although there is no consensus on the necessity and effectiveness of this approach. Laboratory research indicates that, compared to healthy enamel, the use of self-etching primers can favor the bond strength of the bracket to the tooth under shear forces.^33^ ^-^ ^37^

In addition, the orthodontist must consider the previous treatments carried out, ranging from minimally invasive approaches, such as enamel microabrasion and the use of resin infiltrants, to restorative treatments with composite resin or ceramics. Although it is not yet clear how these treatments can influence orthodontic treatment, it is recommended that, where possible, they should be carried out after orthodontics.^38^ ^,^ ^39^ ^,^ ^40^

In MIH, one of the treatment decisions may involve extraction and orthodontic treatment. The decision of extraction in cases with MIH and largely compromised clinical crowns of permanent first molars should be conducted together with an orthodontist.^41^ First molar extraction during the mixed dentition will allow the mesial displacement of second and third molars, closing the spaces naturally in most cases. The prognosis for complete space closure is higher in the upper arch, compared to the lower arch. In both arches, a mesial tip of second and third molars may require the need for comprehensive orthodontic treatment in the complete permanent dentition. Asymmetrical extractions can also cause a midline deviation, and a modified lingual arch can be used to avoid these collateral effects. 

The ideal timing for early extraction with poor prognosis is the mixed dentition between 8 and 10 years of age, when the furcation of mandibular permanent second molars is already observed in the panoramic radiograph.^42^ The timing is more important for the mandibular arch, in which the success rate of complete space closure is decreased, compared to the maxillary arch. The ideal cases are patients with an absence of sagittal maxillomandibular discrepancies and a Class I interarch relationship. The tooth germ of the third molars should be present in the hemi-arch where the extraction is planned. The selection criteria are summarized in Table 1.

**Table 1:** What to consider before interceptive extraction of the first permanent molar in cases of MIH?

POOR PROGNOSIS OF THE COMPROMISED FIRST PERMANENT MOLAR	Severe MIH cases with low survival rate for restorations or recurrent fractures
IDEAL TIMING	Mixed dentition from 8 to 10 years of age, before the eruption of the second premolars
ANTEROPOSTERIOR RELATIONSHIP	Ideally patients with normal occlusion or Class I malocclusion
PRESENCE OF THIRD MOLARS	Third molars must be developing at the time of first permanent molar extractions, once space closure depends on the spontaneous movement of second and third molars

Regarding enamel hypoplasia, it is essential to observe the type and especially the location of the defect. Most hypoplastic lesions are located on the buccal surface of the teeth and, although it is a defect in which enamel maturation occurs as expected, adhesion and retention of orthodontic accessories can be compromised, depending on the extent and irregularity of the affected area. In addition, the morphology of this defect can favor the accumulation of biofilm, which is naturally intensified using fixed appliances, increasing the risk of developing carious lesions.^4^

Since hypoplastic defects can also affect the aesthetics of teeth and lead to hypersensitivity, prior desensitization and the type of material used in the restorative procedure must be considered. In addition, as these are quantitative defects, minimally invasive treatments may not be effective, requiring restorative planning with direct or indirect restorations.^43^ ^-^ ^45^

### ORTHODONTICS AND ENAMEL DEFECTS: PARTICULARITIES AND ADDITIONAL CARE

One of the biggest challenges related to the orthodontic treatment of patients with DED is the lack of robust scientific evidence on the subject. There are major limitations including methodological limitations (e.g., studies with moderate/low-quality of evidence), and most studies are focused on a laboratory approach. Therefore, carrying out clinical trials becomes essential for a more in-depth understanding of the care required during the practice of an orthodontist when challenged by enamel defects. So, make any projection to clinical approach deserves attention and care.

It is also known that the effectiveness and quality of orthodontic treatment depends not only on the professional’s expertise, but also on the choice of materials used, the type of treatment, as well as the type of bracket (metallic or ceramic), the techniques, the patient collaboration and adhesion of accessories to the dental substrate. In defects such as MIH and fluorosis, the etch-and-rise technique using 37% phosphoric acid and adhesive (e.g. Single Bond - 3M, Clearfill Tri S Bond) is effective and has a high degree of evidence.^46^ ^,^ ^47^ Another possible approach in qualitative enamel defects, as previously mentioned, is the deproteinization of the enamel with 5.25% sodium hypochlorite for 1 minute, which can help in the adhesion of the restorative material or orthodontic bracket to the defective enamel.^28^ ^,^ ^29^

The literature also mentions other resources to improve adhesion, such as increasing the acid etching time or using acids in higher concentrations. However, these practices can intensify enamel demineralization, potentially causing damage, and their effectiveness is not yet fully proven. Furthermore, the use of lasers or diamond burs to create microretentions in the enamel or restorative material can promote greater infiltration of the adhesive and orthodontic resin, contributing to more efficient adhesion of accessories to teeth.^44^ ^,^ ^45^ ^,^ ^48^ ^,^ ^49^

The experience of pain during bracket removal may also be reported by patients. In the case of enamel defects, it is essential to treat hypersensitivity before, during, and after treatment, however the technique chosen for removing the device must also be evaluated, aiming for less discomfort.^51^ Removal of the device, especially fixed accessories such as brackets, tubes and bands, and residual resinous material, must be careful and safe, aiming not to cause additional damage to the defective enamel structure.^26^

Additionally, regarding the type of material used for the adhesion of orthodontic accessories, it is possible to choose between glass ionomer cement (GIC) and composite resins. However, the longevity of brackets cemented with composite resins is superior, compared to those cemented with GIC. The removal of brackets, tubes, bands, and remaining material must be carried out with caution, using the Wing removal technique, followed by finishing with multi-laminated carbide drills and Sof-Lex discs, which provide greater smoothness to the surface and reduce risks of fractures in both the enamel and the restorative material.^51^ ^,^ ^52^

In addition to the above-mentioned limitations and challenges, the need for preventive strategies in orthodontic treatment is also highlighted, especially in patients with DDE. The use of toothpaste with a higher concentration of fluoride (5000ppm of fluoride) and mouthwashes can be indicated in a complementary way in the patient’s routine, aiming to control plaque, reducing the risk of developing cavities, as well as in the treatment of gingivitis. The professional must evaluate the indication and need for an individualized prescription, also considering whether the patient has other oral conditions such as the presence of hypersensitivity.^53^ ^,^ ^54^ ^,^ ^55^

Therefore, orthodontic treatment should preferably be performed by a multidisciplinary team, integrating professionals from different specialties. Interceptive measures are recommended for patients with orthopedic demands, considering the dental substrate and treatment predictions, with prior planning of treatment expectations and limitations with the family. Regardless of the patient’s age group, it is essential to promote preventive care to avoid periodontal changes and cavities, factors that can impact orthodontic treatment. Therefore, the orthodontist must be familiar with the diagnosis of DED and consider them in their planning.

**Figure 3:**
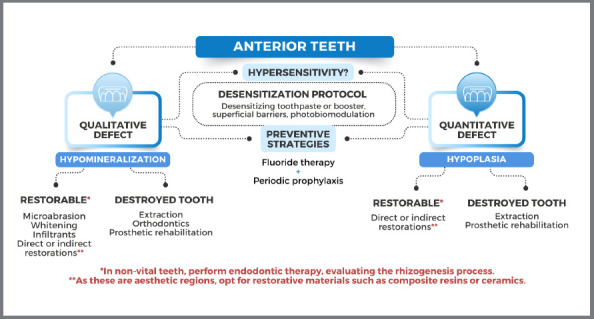
Treatment options for hypomineralized / hypoplastic anterior teeth.

**Figure 4:**
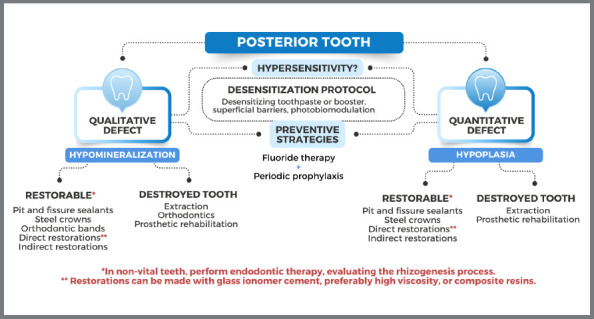
Treatment options for hypomineralized / hypoplastic posterior teeth.

To support the clinical decisions, schematic diagrams were created, to provide proper and feasible solutions to clinicians (Figs. 3 and 4). 

## CONCLUSION

The task of performing orthodontic treatment is one that deserves planning. When enamel defects are present, this task becomes even more challenging. It is therefore necessary for orthodontists to keep up to date on this topic, so that they can provide their patients with the most recent information with good quality evidence. Studies with more representative samples and clinical power are encouraged to provide a more reliable basis for orthodontic practice in these specific cases. 
